# Accelerating the clinical development of protein-based vaccines for malaria by efficient purification using a four amino acid C-terminal ‘C-tag’

**DOI:** 10.1016/j.ijpara.2016.12.001

**Published:** 2017-06

**Authors:** Jing Jin, Kathryn A. Hjerrild, Sarah E. Silk, Rebecca E. Brown, Geneviève M. Labbé, Jennifer M. Marshall, Katherine E. Wright, Sandra Bezemer, Stine B. Clemmensen, Sumi Biswas, Yuanyuan Li, Aadil El-Turabi, Alexander D. Douglas, Pim Hermans, Frank J. Detmers, Willem A. de Jongh, Matthew K. Higgins, Rebecca Ashfield, Simon J. Draper

**Affiliations:** a The Jenner Institute, University of Oxford, Old Road Campus Research Building, Oxford OX3 7DQ, UK; b Department of Biochemistry, University of Oxford, South Parks Road, Oxford OX1 3QU, UK; c Thermo Fisher Scientific, J.H. Oortweg 21, 2333 CH Leiden, Netherlands; d ExpreS^2^ion Biotechnologies, SCION-DTU Science Park, Agern Allé 1, Hørsholm DK-2970, Denmark

**Keywords:** Malaria, Vaccine, *Plasmodium falciparum*, RH5, Blood-stage, Protein purification, Biomanufacture

## Abstract

Development of bespoke biomanufacturing processes remains a critical bottleneck for translational studies, in particular when modest quantities of a novel product are required for proof-of-concept Phase I/II clinical trials. In these instances the ability to develop a biomanufacturing process quickly and relatively cheaply, without risk to product quality or safety, provides a great advantage by allowing new antigens or concepts in immunogen design to more rapidly enter human testing. These challenges with production and purification are particularly apparent when developing recombinant protein-based vaccines for difficult parasitic diseases, with *Plasmodium falciparum* malaria being a prime example. To that end, we have previously reported the expression of a novel protein vaccine for malaria using the ExpreS^2^*Drosophila melanogaster* Schneider 2 stable cell line system, however, a very low overall process yield (typically <5% recovery of hexa-histidine-tagged protein) meant the initial purification strategy was not suitable for scale-up and clinical biomanufacture of such a vaccine. Here we describe a newly available affinity purification method that was ideally suited to purification of the same protein which encodes the *P. falciparum* reticulocyte-binding protein homolog 5 – currently the leading antigen for assessment in next generation vaccines aiming to prevent red blood cell invasion by the blood-stage parasite. This purification system makes use of a C-terminal tag known as ‘C-tag’, composed of the four amino acids, glutamic acid – proline – glutamic acid – alanine (E-P-E-A), which is selectively purified on a CaptureSelect™ affinity resin coupled to a camelid single chain antibody, called NbSyn2. The C-terminal fusion of this short C-tag to *P. falciparum* reticulocyte-binding protein homolog 5 achieved >85% recovery and >70% purity in a single step purification directly from clarified, concentrated Schneider 2 cell supernatant under mild conditions. Biochemical and immunological analysis showed that the C-tagged and hexa-histidine-tagged *P. falciparum* reticulocyte-binding protein homolog 5 proteins are comparable. The C-tag technology has the potential to form the basis of a current good manufacturing practice-compliant platform, which could greatly improve the speed and ease with which novel protein-based products progress to clinical testing.

## Highlights

• Fusion of a four amino acid ‘C-tag’ allows purification of a PfRH5 malaria vaccine.
• Overall process yield of 40–45% and very high product purity (>99%) was achieved.
• His6-tagged and C-tagged PfRH5 are conformational and bind to basigin.
• C-tag will facilitate the clinical translation of difficult-to-produce antigens.

## Graphical abstract

**Figure:**
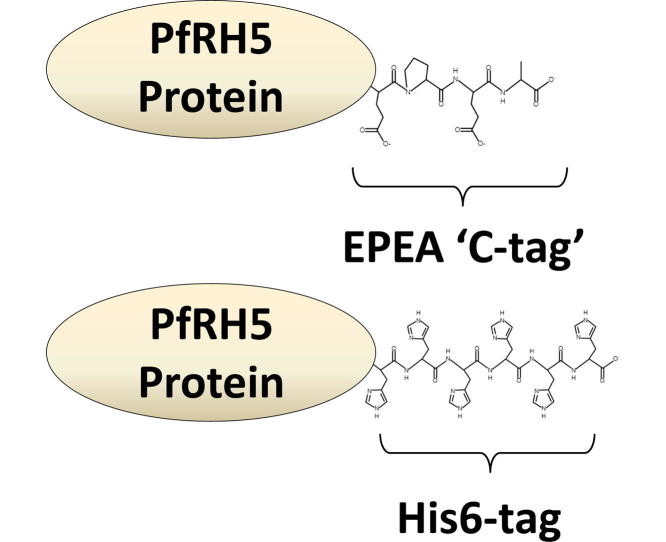

## 1 Introduction

The production of recombinant antigen remains central to the development of many types of subunit vaccines, and especially for those seeking to induce antibody (Draper et al., 2015). Such antigen may take numerous forms, ranging from a relatively simple peptide to soluble monomeric protein through to more complex oligomeric scaffolds (Li et al., 2016) or larger virus-like particles (VLPs) (Wu et al., 2015, Brune et al., 2016). Following purification, the classical approach to antibody induction by subunit vaccination has been the delivery of the protein or VLP antigen formulated in a chemical adjuvant (Coler et al., 2009, de Cassan et al., 2011), with notable success in humans including hepatitis B virus surface antigen (HBsAg) and bacterial toxoids (tetanus and diphtheria). These approaches are further exemplified by ongoing efforts to develop a highly effective vaccine against infection, disease or transmission caused by the *Plasmodium falciparum* human malaria parasite (Halbroth and Draper, 2015). In this case multiple stages of the parasite’s complex lifecycle are susceptible to functional antibodies – including sporozoites, merozoites, infected red blood cells, gametocytes and sexual stages within the mosquito.

Current subunit vaccine strategies are seeking to improve on the modest levels of efficacy reported for the RTS,S/AS01 malaria vaccine – based on the recombinant HBsAg VLP technology and which targets the pre-erythrocytic circumsporozoite antigen (Rts, 2015). One leading approach will involve future trialling with a multi-antigen, multi-stage formulation by combining with other effective vaccine components against the pathogenic asexual blood-stage of infection (Goodman and Draper, 2010) and/or the subsequent sexual/mosquito stages (Nikolaeva et al., 2015). The blood-stage vaccine component would seek to protect against death and clinical disease, whilst contributing to reduced transmission through control and clearance of blood-stage parasitemia. The mainstay approach in this arena has involved targeting merozoite proteins involved in the red blood cell (RBC) invasion process. Although historical candidates have suffered from substantial levels of polymorphism leading to induction of strain-specific antibody responses (Remarque et al., 2008), a new generation of targets are being identified which are relatively highly conserved and yet susceptible to neutralising antibodies raised by vaccination. Currently the most advanced of these candidates is the *P. falciparum* reticulocyte-binding protein homolog 5 (PfRH5) (Drew and Beeson, 2015). Antibodies raised by vaccination of animals can cross-inhibit all *P. falciparum* lines and field isolates tested to date (Douglas et al., 2011, Williams et al., 2012, Bustamante et al., 2013, Reddy et al., 2014), also with higher efficiency than other historical target antigens (Williams et al., 2012). Importantly, PfRH5 is reported to be essential (Hayton et al., 2008, Baum et al., 2009), and forms a critical non-redundant interaction with its receptor, basigin (CD147), during invasion (Crosnier et al., 2011). Moreover, the relatively high degree of PfRH5 sequence conservation is associated with low-level immune pressure following natural infection (Douglas et al., 2011, Villasis et al., 2012, Tran et al., 2014), as well as functional constraints linked to basigin binding and host RBC tropism (Hayton et al., 2008, Hayton et al., 2013, Wanaguru et al., 2013). Vaccination of *Aotus* monkeys also showed significant efficacy against a stringent heterologous strain blood-stage *P. falciparum* challenge, where protection was strongly associated with anti-PfRH5 serum IgG antibody concentration and in vitro growth inhibition activity (GIA) measured using purified IgG (Douglas et al., 2015).

The earliest vaccination studies with PfRH5 used fragments of the antigen made in *Escherichia coli* that failed to induce functional antibodies (Rodriguez et al., 2008, Baum et al., 2009). Consequently, efforts focussed on protein immunogens based on the full-length PfRH5 sequence which resulted in functional neutralising antibodies (Douglas et al., 2011, Bustamante et al., 2013, Reddy et al., 2014), some of which are known to block the PfRH5-basigin interaction (Douglas et al., 2014). However, despite these successes, it proved particularly problematic to develop a process that is scalable and compliant with current good manufacturing practice (cGMP) and which could enable production of a batch of full-length PfRH5 protein for use in clinical trials. Recently, we reported the production of soluble full-length PfRH5 protein using a cGMP-compliant platform called ExpreS^2^ (Dyring, 2011), based on a *Drosophila melanogaster* Schneider 2 (S2) stable cell line system (Wright et al., 2014, Hjerrild et al., 2016). Full-length PfRH5 protein was expressed from stable cell lines and purified using a C-terminal hexa-histidine (His6) tag, and induced functional antibodies following immunisation of rabbits. However, despite successful expression in this heterologous system, up to four purification steps were required, resulting in high (>95%) purity of the final PfRH5 protein but a low overall process yield, typically <5% recovery (Hjerrild et al., 2016). Consequently this purification strategy was not suitable for scale-up and clinical biomanufacture of a PfRH5 protein vaccine.

Here we describe a newly available affinity purification method that was ideally suited to purification of the PfRH5 protein. This system makes use of a C-terminal tag known as ‘C-tag’, composed of the four amino acids (aa), glutamic acid – proline – glutamic acid – alanine (E-P-E-A), which is selectively captured on a resin coupled to a camelid single chain antibody, termed NbSyn2 and specific for this short sequence. NbSyn2 was originally raised by immunisation of a dromedary with alpha-synuclein (De Genst et al., 2010) and further developed into a CaptureSelect™ affinity resin by BAC B.V. in the Netherlands (now Thermo Fisher Scientific). This resin can be produced to be suitable for use in clinical and commercial biomanufacture. Notably, multiple different single chain antibody-based affinity resins are now available for cGMP manufacture, and the first product purified in this manner, an adeno-associated virus gene therapy product called alipogene tiparvovec (Glybera®) for lipoprotein lipase deficiency, has been licenced in Europe (Wang et al., 2011, Wang et al., 2015). Here, C-terminal fusion of this short C-tag to PfRH5 achieved >85% recovery and >70% purity in a single step purification directly from clarified, concentrated S2 cell supernatant under mild conditions. The purification and biochemical analysis of the PfRH5 protein is reported, whilst a functional analysis of the antibodies induced following immunisation of rabbits shows comparable immunogenicity to His6-tagged antigen. Our data suggest that the C-tag technology could form the basis of a cGMP-compliant platform that will improve the speed and ease of process development for the biomanufacture of novel protein-based products.

## 2 Materials and methods

### 2.1 Design and cloning of PfRH5 protein vaccines

All chemicals were purchased from Sigma–Aldrich, UK unless otherwise specified. The design of the PfRH5 protein with C-terminal His6 tag has been described elsewhere, where it was reported as variant version 1.0 (Hjerrild et al., 2016). In brief, the protein encodes the full-length ectodomain of the PfRH5 antigen (aa E26-Q526) based on the sequence of the 7G8 laboratory-adapted *P. falciparum* parasite line, and all four putative N-linked glycosylation sequons (N-X-S/T) were mutated Thr to Ala – as performed for a previous PfRH5 protein vaccine produced in mammalian HEK293 cells and tested in rabbits (Bustamante et al., 2013) and *Aotus* monkeys (Douglas et al., 2015). A synthetic gene was designed based on the above 7G8 sequence for PfRH5 and codon-optimised for expression in *D. melanogaster* (GeneArt, Thermo Fisher Scientific, Germany). The construct also contained a Kozak sequence (GCC ACC) at the 5′ end, an N-terminal 18 aa Ig heavy chain binding protein (BiP) insect signal peptide (MKLCILLAVVAFVGLSLG) and a C-terminal His6 tag. This gene insert was subcloned into the pExpreS^2^-1 plasmid allowing for Zeocin selection (ExpreS^2^ion Biotechnologies, Denmark). Subsequently, the C-terminal His6 tag coding sequence was replaced within the plasmid with that encoding the C-tag – four amino acids E-P-E-A.

### 2.2 Generation of the polyclonal Drosophila S2 stable cell line

The generation of a stable cell line expressing PfRH5-His6 has been previously described in detail (Hjerrild et al., 2016). Identical methods were used here to generate the stable cell line expressing the PfRH5-C-tag. Briefly, the ExpreS^2^ *Drosophila* S2 cell line was used (ExpreS^2^ion Biotechnologies, Denmark) and cells were cultured in EX-CELL 420 media +10% FBS. Cells were seeded at 2 × 10^6^ cells/mL in EX-CELL 420 media without FBS, and transfected with plasmid DNA using ExpreS^2^ Insect-TRx1 transfection reagent (ExpreS^2^ion Biotechnologies). Zeocin selection was applied from day 2 through to day 26 post-transfection. On day 26, cells were transferred into a 125 mL shake flask with the addition of fresh EX-CELL 420 media +10% FBS. The cells were then passaged once by centrifugation to remove any residual Zeocin, before freezing.

### 2.3 SDS–PAGE and western blots

Samples were prepared in 4× Laemmli buffer containing DTT as the reducing agent (BioRad, UK) and heated to 95 °C for 10 min. Electrophoresis was performed on a Novex 4–12% Bis-Tris gel in MES SDS running buffer (Thermo Fisher Scientific, UK) at 200 V for 35 min. The gels were then stained with Quick Coomassie (Generon, UK) for total protein, or for western blotting, were further transferred to a nitrocellulose membrane using a Trans-blot turbo transfer system (BioRad). Blots were processed in an iBind Western device (Thermo Fisher Scientific) using polyclonal anti-PfRH5 rabbit serum (Douglas et al., 2011). Product purity and recovery were estimated using densitometry analysis by ImageJ software (Schneider et al., 2012).

### 2.4 Recombinant PfRH5 protein purification

Growth conditions of stably transfected *Drosophila* S2 cells expressing His6-tagged PfRH5 and C-tagged PfRH5 were identical to those described previously (Hjerrild et al., 2016). Clarified supernatant from a day 4 batch culture was concentrated 15–20-fold and the buffer exchanged using a Tangential Flow Filtration (TFF) system fitted with a Pellicon 3 Ultracel 10 kDa membrane (Merck Millipore, UK).

For both constructs, the purification scheme consisted of capture step chromatography followed by polishing Size Exclusion Chromatography (SEC) performed on an AKTA Pure 25 system (GE Healthcare, UK). Specifically for His6-tagged PfRH5, concentrated supernatant in the equilibrium and wash buffer (20 mM Tris–HCl, 300 mM NaCl, 5 mM imidazole, pH 8.0) were applied to a cobalt-based immobilised Metal Affinity Chromatography (IMAC) – Hitrap TALON crude column (GE Healthcare). Elution took place at an elevated imidazole concentration of 150 mM. The final polishing SEC was achieved isocratically on a Superdex 200 16/60 PG column (GE Healthcare) in 20 mM Tris–HCl, 150 mM NaCl, pH 7.4 (TBS). Fractions corresponding to PfRH5 were pooled and stored at −80 °C until further analysis. For C-tagged PfRH5 protein, while the polishing SEC remained the same, a CaptureSelect™ C-tag affinity column (Thermo Fisher Scientific) was employed instead of TALON in the capture step. The C-tag column was conditioned in TBS before sample application, then washed in TBS and eluted in 20 mM Tris–HCl, 2 M MgCl_2_, pH 7.4.

### 2.5 Surface plasmon resonance (SPR)

The production of recombinant basigin in Origami B (DE3) *E. coli* cells has been previously described (Wright et al., 2014). A section of the basigin gene encoding Ig domains 1 and 2 of the short isoform (aa 22–205) was cloned with an N-terminal His6 tag followed by a tobacco etch virus (TEV) protease cleavage site. TEV cleavage leaves an additional glycine at the N-terminus from the cleavage site. SPR experiments were carried out using a BIAcore T200 instrument (GE Healthcare). Experiments were performed at 20 °C in 10 mM HEPES (pH 7.4), 150 mM NaCl, 3 mM EDTA, 0.005% Tween-20, 2 mg/mL of dextran, and 1 mg/mL of salmon sperm DNA. Basigin was immobilised on a CM5 chip (GE Healthcare) by amine coupling (GE Healthcare kit) to a total of 950 Response Units (RU). A concentration series of each PfRH5 variant protein (a twofold dilution series from 2 μM) was injected over the basigin-coated chip for 120 s at 30 μL/min, followed by a 300 s dissociation time. The chip surface was then regenerated for 30 s with 2 M NaCl. Specific binding of the PfRH5 protein was obtained by subtracting the response from a blank surface from that of the basigin-coated surface. The kinetic sensorgrams were fitted to a global 1:1 interaction model, allowing determination of the dissociation constant, K_D_, using BIAevaluation software 1.0 (GE Healthcare, UK).

### 2.6 Monoclonal antibody (mAb) ELISA

The generation of eight PfRH5-specific mouse mAbs has been previously described (Douglas et al., 2014). Purified mAb was coated at 5 μg/mL with 50 μL/well onto a Maxisorp plate (Thermo Fisher Scientific) and incubated at 4 °C overnight. The following day, the plates were washed six times with PBS/0.05% Tween-20 (PBS/T), before blocking with 150 μL/well of Casein Blocker (Thermo Fisher Scientific) at room temperature (RT) for 1 h. After washing again six times in PBS/T, PfRH5 proteins were loaded (50 μL in triplicate) on to the plate at four dilutions in Casein Blocker (800, 200, 50 and 12.5 ng/mL) and incubated at RT for 2 h. After a further wash, plates were incubated with polyclonal anti-PfRH5 rabbit serum (Douglas et al., 2011) diluted 1:1000 in Casein Blocker, using 50 μL/well at RT for 1 h. After a further wash, plates were incubated with goat anti-rabbit IgG alkaline phosphatase diluted 1:5000 in Casein Blocker, using 50 μL/well at RT for 1 h. After a final six washes in PBS/T, followed by two washes in PBS, plates were developed by addition of *p*-nitrophenyl phosphate substrate diluted in diethanolamine buffer (Thermo Fisher Scientific). The O.D. at 405 nm (OD_405_) was read using a Tecan Infinite F50 microplate reader (Tecan, Switzerland).

### 2.7 Rabbit immunisation

Animal experiments were approved by the University of Oxford Animal Welfare and Ethical Review Body, UK, and performed in accordance with all applicable regulations. Rabbit protein immunizations were carried out by Biogenes (Germany). ZiKa rabbits (*n* = 4/group) were immunised i.m. with 20 μg of protein on day 0 formulated in FCA, followed by two booster immunizations i.m. on days 28 and 56 with the same dose of protein formulated in incomplete Freund’s adjuvant. Serum was collected pre-immunisation (day 0) and 2 weeks after the final immunisation on day 70 and shipped frozen.

### 2.8 Anti-PfRH5 serum IgG ELISA

Anti-PfRH5 serum IgG responses were measured by ELISA using previously described methodology (Hjerrild et al., 2016). In this case, the C-tagged PfRH5 protein used was based on the 3D7 clone sequence – differing by only one amino acid from the 7G8 sequence proteins tested here (cysteine at position 203 as opposed to tyrosine). Importantly, small numbers of polymorphic residues that differ between PfRH5 allelic variants have been shown to not have a detectable impact on ELISA cross-reactivity (Bustamante et al., 2013). A standardised ELISA format was used (Miura et al., 2008, Sheehy et al., 2011) including a reference sample generated from high-titer sera from a viral vectored immunised rabbit (Douglas et al., 2011). Test samples were diluted appropriately so that their OD_405_ could be read from the linear part of the reference curve. In order to convert the responses to μg/mL, an affinity-purified reference standard of rabbit anti-PfRH5-specific IgG was prepared as reported elsewhere (Hjerrild et al., 2016) and used to create a conversion factor from the arbitrary ELISA units. Rabbit IgG concentration ELISAs were measured using a standardised ELISA as reported previously (Hjerrild et al., 2016).

### 2.9 Assay of growth inhibition activity (GIA)

Total IgG was purified from rabbit sera using protein G columns (Thermo Scientific Pierce, UK). The *P. falciparum* 3D7 clone and 7G8 laboratory-adapted line were maintained in continuous culture using fresh human blood group O+ erythrocytes at 2% hematocrit and synchronised by two incubations in 5% sorbitol 6–8 h apart. Synchronised trophozoites were adjusted to 0.3% parasitemia for 3D7 or 0.5% for 7G8 and then incubated for 42 h with the various IgG concentrations. Final parasitemia was determined by biochemical determination of parasite lactate dehydrogenase (Miura et al., 2009). Percentage growth inhibition was expressed relative to wells containing IgG from control immunised rabbits (Douglas et al., 2011). The mean of the three replicate wells was taken to obtain the final data for each individual rabbit at each tested IgG concentration. Experiments were performed twice with very similar results.

### 2.10 Production of recombinant α-synuclein

Human α-synuclein coding sequence (aa 1–140 from UniProtKB number P37840-1) preceded by a His6 tag with a TEV protease cleavage site, codon optimised for *E. coli* (GeneArt, Thermo Fisher Scientific), was inserted by restriction enzyme digestion into a prokaryotic expression vector (pET28b, Merck Millipore). Plasmid was transformed into an *E. coli* expression host (BL21(DE3)Star, Thermo Fisher Scientific) and protein induced with 1 mM IPTG at 37 °C for 4 h. Clarified soluble cell lysate was subject to Nickel-ion affinity purification (HisTrap™ Excel, GE Healthcare) following the manufacturer’s instructions. This was followed by protease cleavage for removal of the His6-tag (TEV protease containing His6-tag was a kind gift from the Structural Genomics Consortium, Oxford, UK) and further affinity chromatography, effectively removing the cleaved tag and protease, yielding α-synuclein protein (approximately 90% pure, from Coomassie stained SDS–PAGE).

### 2.11 Anti-tag ELISAs

Streptavidin plates (Thermo Fisher Scientific) were coated at 4 °C overnight in Dulbecco’s PBS with N-terminal biotin-peptides (NeoScientific, USA) at 10 μg/mL. Plates were washed with PBS/T and blocked in 1% BSA in PBS/T for 1 h at RT. Plates were washed again with PBS/T and blocked with 10 μg/mL of free biotin for 1 h at RT. Rabbit serum samples were diluted 1:100 in 0.1% BSA in PBS/T and added in triplicate following another wash step. Anti-C-tag-biotin (Thermo Fisher Scientific) at 1 μg/mL in PBS/T was included as a positive control. Plates were incubated at RT for 2 h and then washed prior to addition of alkaline phosphatase-labelled goat anti-rabbit IgG (whole molecule) to sample wells or alkaline phosphatase-labelled streptavidin added to control wells. Both secondary antibodies were added at 1:1000 in 0.1% BSA in PBS/T and left to incubate for 1 h at RT. Bound antibodies were detected as for the mAb ELISA and OD_405_ read using a Tecan Infinite F50 microplate reader (Tecan).

For protein, Maxisorp plates (Thermo Fisher Scientific) were coated at 4 °C overnight in Dulbecco’s PBS with recombinant α-synuclein protein or Pfs25-His6 (Li et al., 2016) at 2 μg/mL. Plates were washed with PBS/T, blocked in Casein block solution (Pierce) for 1 h at RT, and washed again prior to addition of rabbit serum samples as for the peptide ELISAs but diluted at 1:100 or 1:300 in Casein block solution. Anti-C-tag-biotin at 1 μg/mL or mouse anti-histidine tag reagent (clone AD1.1.10, Abd Serotec, UK) used at 1 μg/mL in Casein block solution were included as positive controls. Plates were incubated at RT for 2 h and then washed prior to addition of alkaline phosphatase-labelled goat anti-rabbit IgG (whole molecule) to sample wells, alkaline phosphatase-labelled anti-mouse IgG (whole molecule) to the anti-histidine-positive control wells, or alkaline phosphatase-labelled streptavidin to the anti-C-tag control wells. All secondary antibodies were added at 1:1000 in Casein block solution and left to incubate for 1 h at RT. Bound antibodies were detected as for the peptide ELISAs.

### 2.12 Statistical analysis

Data were analysed using GraphPad Prism version 5.04 for Windows (GraphPad Software Inc., California, USA). For the non-linear least squares regression, the equation: Y = Bottom + (Top–Bottom)/(1 + 10^((LogEC_50_ − X) * HillSlope)) was used with four parameter curve and log_10_ transformed ELISA data, constrained at the top to <100% and at the bottom to >0% GIA.

## 3 Results

### 3.1 Generation of a polyclonal Drosophila S2 stable cell line expressing the PfRH5-C-tag protein vaccine

**Fig. 1:**
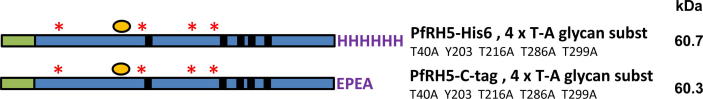
*Plasmodium falciparum* reticulocyte-binding protein homolog 5 vaccine constructs showing a schematic of *P. falciparum* reticulocyte-binding protein homolog 5 proteins. Both constructs encoded from the N-terminus: a BiP insect signal peptide (green) followed by the ectodomain of *P. falciparum* reticulocyte-binding protein homolog 5 (amino acids 26–526) (blue) followed by a C-terminal tag – either hexa-histidine or the glutamic acid – proline – glutamic acid – alanine C-tag. The proteins were based on the *P. falciparum* 7G8 strain sequence which has tyrosine (Y) at position 203 (and not cysteine as in the 3D7 clone reference genome sequence) (yellow circle). The other cysteine residues in *P. falciparum* reticulocyte-binding protein homolog 5 are indicated by small black boxes (C224, C317, C329, C345 and C351). Threonine (T) to alanine (A) substitutions to remove N-linked glycan sequons are indicated by red asterisks. The predicted molecular weight for each protein is indicated.

**Fig. 2:**
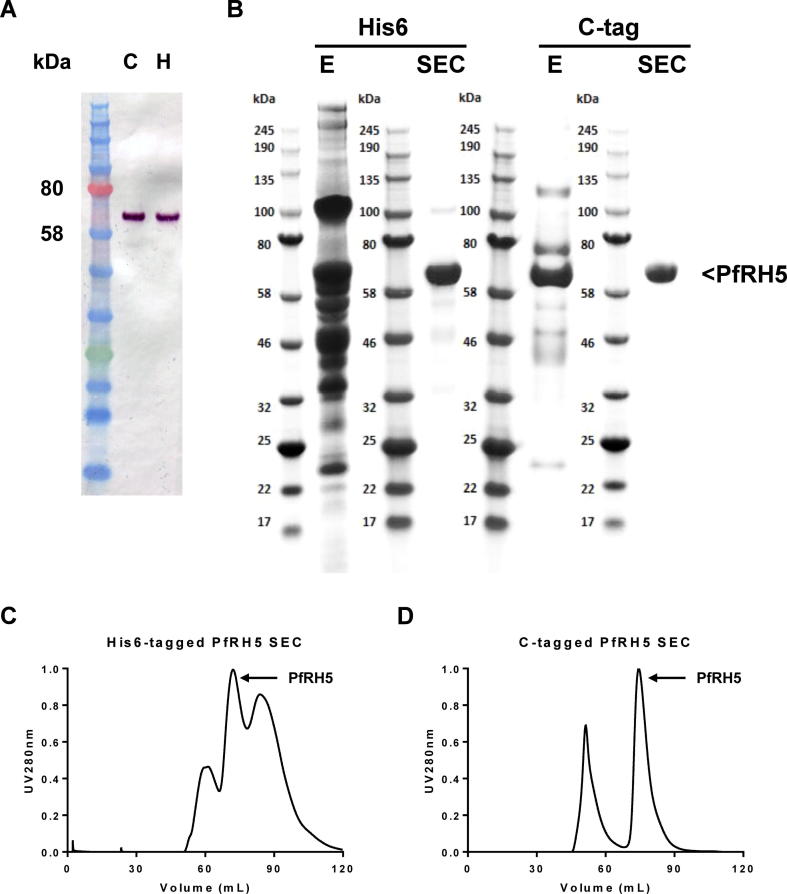
Purification of *Plasmodium falciparum* reticulocyte-binding protein homolog 5 proteins. (A) Supernatant samples from batch cultures were run on SDS–PAGE under reducing conditions and western blotting performed with polyclonal anti-*P. falciparum* reticulocyte-binding protein homolog 5 rabbit serum. C, glutamic acid – proline – glutamic acid – alanine C-tagged protein; H, hexa-histidine-tagged protein. (B) Purity assessment of hexa-histidine-tagged *P. falciparum* reticulocyte-binding protein homolog 5 TALON column eluate (labelled E); hexa-histidine-tagged *P. falciparum* reticulocyte-binding protein homolog 5 Size Exclusion Chromatography eluate; C-tagged *P. falciparum* reticulocyte-binding protein homolog 5 C-tag eluate (labelled E); and C-tagged *P. falciparum* reticulocyte-binding protein homolog 5 Size Exclusion Chromatography eluate by SDS–PAGE under reducing conditions. (C) UV 280 nm absorbance chromatogram of hexa-histidine-tagged and (D) C-tagged *P. falciparum* reticulocyte-binding protein homolog 5 polishing SEC steps.

We have previously reported the production of a full-length PfRH5 protein vaccine with C-terminal His6 tag using a stable *Drosophila* S2 stable cell line (Hjerrild et al., 2016). Here we produced a second version of this gene in an identical manner, replacing the His6 tag with the four aa C-tag (EPEA) (Fig. 1). This gene was subcloned into the pExpreS^2^-1 plasmid allowing for Zeocin selection in transfected *Drosophila* S2 cells. Initial studies confirmed expression of the C-tagged PfRH5 protein following transient transfection (data not shown). Subsequently, a stable cell line was generated over a 26 day period using Zeocin selection as described previously (Hjerrild et al., 2016). The cell line was frozen and supernatants harvested for analysis by western blot. PfRH5-C-tag protein was visible by western blot at the expected size of approximately 60 kDa, comparable with PfRH5-His6 protein (Fig.2A).

### 3.2 Purification of PfRH5 protein vaccines from polyclonal *Drosophila* S2 stable cell line supernatants

Our previous attempts to purify PfRH5-His6 tagged protein resulted in high (>95%) purity but low overall process yields averaging less than 5% on most runs – this was not suitable to progress towards vaccine biomanufacture (Hjerrild et al., 2016). This process utilised a concentration and buffer exchange step by TFF, followed by Nickel-IMAC (Ni-IMAC), a Concanavalin A (Con A) 4B column in flow-through mode, and a polishing step using SEC. The heaviest losses were observed during the Ni-IMAC step. Here we sought to further optimise this process, through use of an alternative cobalt-based TALON column (IMAC step) to capture the His6-tagged PfRH5 protein, versus immunoaffinity purification with the C-tag.

The PfRH5 proteins were purified from four-day batch cultures in shaker flasks. Both supernatants were concentrated and buffer exchanged using TFF, typically giving 80–90% process recovery (Table 1), consistent with our earlier work (Hjerrild et al., 2016). PfRH5-His6 tagged protein in IMAC equilibration buffer was purified using the TALON column, followed by a polishing SEC, with step yields of approximately 60% and 50%, respectively. This resulted in an overall process yield of approximately 25% on average (Table 1). Use of the cobalt-based TALON column thus represented a substantial improvement over the previous protocols using Ni-IMAC, however, it was clear from analysis by SDS–PAGE that the TALON eluate still contained numerous contaminants. Following the capture step, PfRH5 purity was only 20%, although the subsequent SEC step was able to improve the purity to over 88% (Fig.2B, C).

**Table 1:** *Plasmodium falciparum* reticulocyte-binding protein homolog 5 purification process yield for C-terminal hexa-histidine-tagged and C-tagged (glutamic acid – proline – glutamic acid – alanine) constructs. Representative data for the amount of *P. falciparum* reticulocyte-binding protein homolog 5 protein recovered after each step of the purification process. Protein recovery was estimated using densitometry analysis of SDS–PAGE.

Process yield (after)	His6-tagged construct	C-tagged construct
Culture supernatant	100%	100%
TFF	82.1%	91.0%
Affinity chromatography	52.5%	77.4%
SEC	25.5%	43.3%

TFF; Tangential Flow Filtration; SEC, Size Exclusion Chromatography.

We continued to compare this purification process with immunoaffinity purification using the C-tagged construct. Following TFF the protein was exchanged into TBS and applied to the C-tag column followed by SEC. Use of the C-tag gave high-level recovery for the capture step (on average 85%) with the eluate showing 72% purity. The SEC polishing step again showed a step yield of approximately 50%, similar to before; this resulted in an overall process yield of 40–45% (Table 1) with very high product purity of >99% (Fig.2B, D).

### 3.3 Biochemical characterization of PfRH5 protein variants

**Fig. 3:**
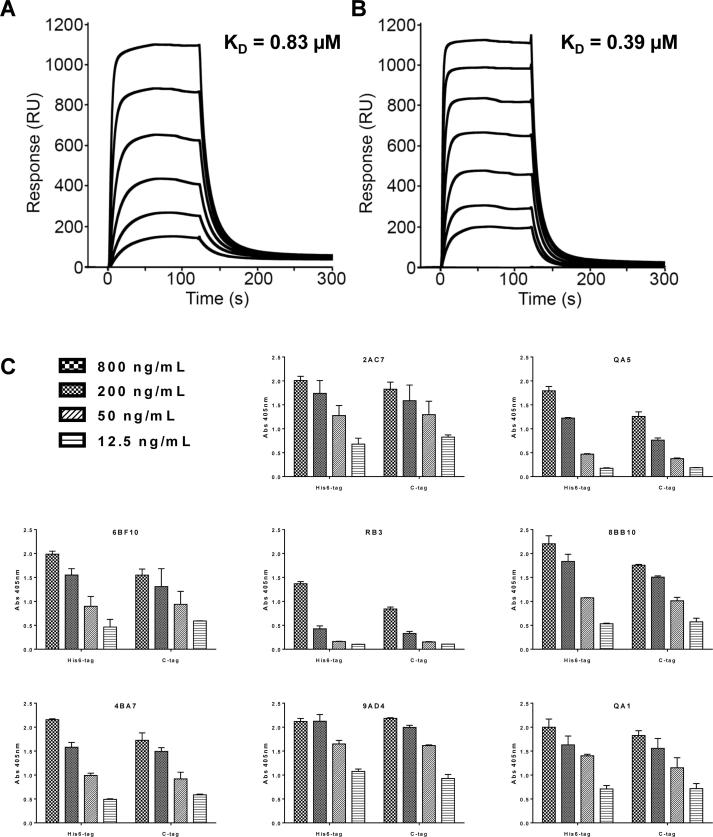
Characterization of *Plasmodium falciparum* reticulocyte-binding protein homolog 5-C-tag (glutamic acid – proline – glutamic acid – alanine) protein. Surface plasmon resonance analysis of the interaction and affinity (K_D_) of (A) C-terminal hexa-histidine-tagged and (B) C-tagged *P. falciparum* reticulocyte-binding protein homolog 5 protein with basigin. RU, Response Units. (C) Capture ELISA using a panel of *P. falciparum* reticulocyte-binding protein homolog 5-specific monoclonal antibodies (2AC7, QA5, 6BF10, RB3, 8BB10, 4BA7, 9AD4, QA1). Both *P. falciparum* reticulocyte-binding protein homolog 5 proteins were tested for binding using a dilution series ranging from 800 ng/mL to 12.5 ng/mL. Each sample was tested in triplicate for each concentration. Bars show the median plus range. Abs, absorbance.

We next assessed the ability of the purified C-tagged protein variant to bind recombinant basigin by SPR. The C-tagged protein bound to basigin with an affinity of 0.39 μM while the His6-tagged protein bound with an affinity of 0.83 μM (Fig.3A, B). These values are close to the reported K_D_ of 1 μM (Crosnier et al., 2011, Bustamante et al., 2013, Wright et al., 2014, Douglas et al., 2015, Hjerrild et al., 2016), although the slightly higher affinity of the C-tagged material indicates there may be a small improvement in protein quality. Both proteins were also recognised in an ELISA assay by a panel of eight previously characterised mouse mAbs (Douglas et al., 2014) (Fig.3C), confirming the presence of each epitope in both proteins. These data confirmed that the C-tagged protein was conformational and capable of binding its receptor, in a comparable manner to the His6-tagged protein.

### 3.4 Immunological analysis of PfRH5 protein variants

**Fig. 4:**
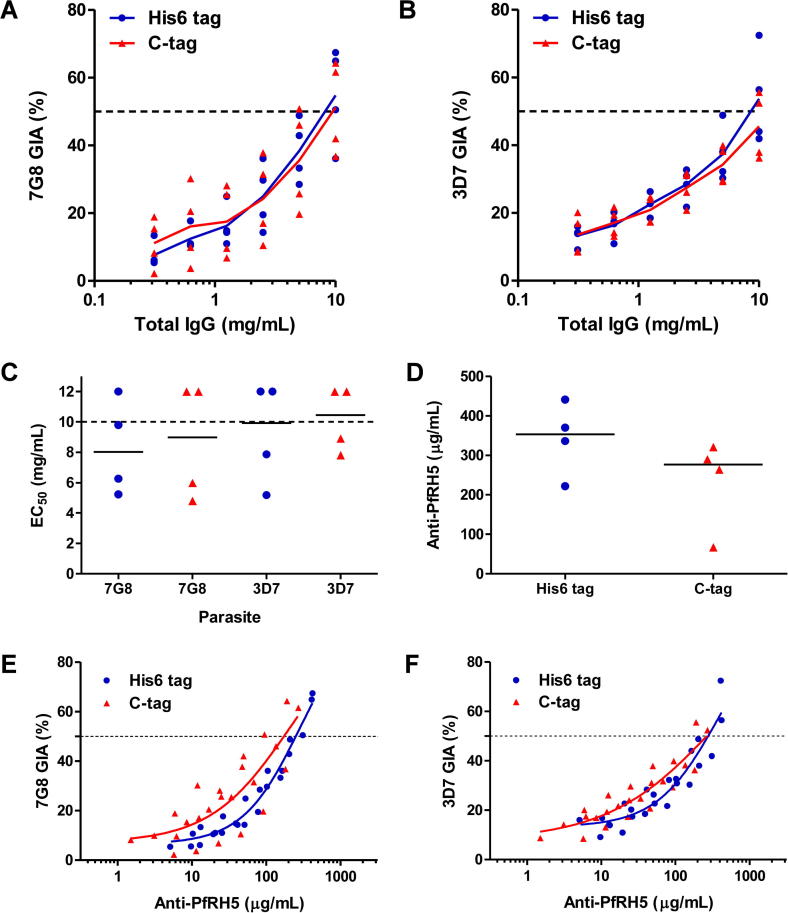
Immunological analysis of *Plasmodium falciparum* reticulocyte-binding protein homolog 5-C-tag protein vaccine. Growth inhibition activity against (A) the *P. falciparum* 7G8 laboratory-adapted parasite line and (B) *P. falciparum* 3D7 clone parasites versus total IgG concentration, with lines connecting data for each group of rabbits (*n* = 4/group) either vaccinated with C-terminal hexa-histidine-tagged, or glutamic acid – proline – glutamic acid – alanine C-tagged *P. falciparum* reticulocyte-binding protein homolog 5 protein. Individual data points are shown plus the line connecting the mean responses. Each GIA value is the mean of triplicate wells tested in the experiment. All GIA experiments were performed twice, with one representative result shown. Dotted line indicates 50% growth inhibition. (C) Total IgG concentrations (mg/mL) that gave 50% growth inhibition (EC_50_) in the assay of growth inhibition activity. Points show the mean result for each rabbit tested in duplicate in two independent experiments. Where individual rabbits did not achieve 50% GIA at the highest tested IgG concentration, i.e. EC_50_ > 10 mg/mL (indicated by the dotted line), these are plotted arbitrarily as 12 mg/mL. Median lines are shown. (D) Anti-*P. falciparum* reticulocyte-binding protein homolog 5 ELISA results are shown quantified in terms of μg/mL. Individual and median results are shown for each group. (E) Dose–response curve fitted to all 7G8 growth inhibition activity versus anti-*P. falciparum* reticulocyte-binding protein homolog 5 antigen-specific antibody concentration data (all IgG dilutions for each rabbit are shown). Dashed horizontal line indicates 50% growth inhibition activity. Non-linear least squares regression line is shown; *r*^2^ = 0.96 for hexa-histidine and 0.69 for C-tag, *n* = 24 for both. (F) Same analysis as (E) against 3D7 parasites; *r*^2^ = 0.87 for hexa-histidine and 0.82 for C-tag, *n* = 24 for both.

In order to test the immunogenicity and functional activity of antibodies induced by the PfRH5 vaccines encoding the C-terminal His6 or C-tag, we immunised rabbits three times with 20 μg of antigen formulated in Freund’s adjuvant prior to serum harvest. IgG was then purified and assessed for in vitro GIA in a single-cycle assay. The levels of functional GIA across the IgG dilution series were comparable for both vaccines against the homologous 7G8 parasite (Fig.4A), and the laboratory reference clone 3D7 (Fig.4B), with no significant differences between the observed IgG concentrations that gave 50% GIA (EC_50_s) (Fig.4C). Each rabbit serum sample was also tested for anti-PfRH5 IgG responses by ELISA, with no significant difference observed between the two groups (Fig.4D) (Mann–Whitney test, *P* = 0.20). Median responses of 353 and 277 μg/mL of PfRH5-specific IgG were observed for the His6 and C-tag groups, respectively.

We next assessed the relationship between anti-PfRH5 antibody responses and functional GIA. The concentration of rabbit total IgG in each serum sample was first assayed by ELISA (median = 13.5 mg/mL; range 8.0–17.6 mg/mL; *n* = 8). Subsequently, the ELISA result for each serum sample (Fig.4D) was normalised according to the serum IgG concentration to give the μg/mL of anti-PfRH5 IgG concentration per mg of total IgG. The GIA data for each individual rabbit were then replotted against the concentration of anti-PfRH5 IgG present in each purified IgG sample tested in the assay (Fig.4E, F). These data showed that GIA was associated with anti-PfRH5 IgG concentration, with a typical sigmoidal relationship, as observed in numerous other studies with other antigens (Miura et al., 2009, Hodgson et al., 2014). The curves and EC_50_s were similar for each vaccine tested against both 7G8 and 3D7 parasites, although the weakest relationship was observed for the C-tagged vaccine tested against 7G8. The other three analyses showed a stronger fit of the data. Overall, these data suggest that the His6- and C-tagged PfRH5 protein vaccines perform similarly in rabbits in terms of both quantitative and qualitative IgG antibody responses.

**Fig. 5:**
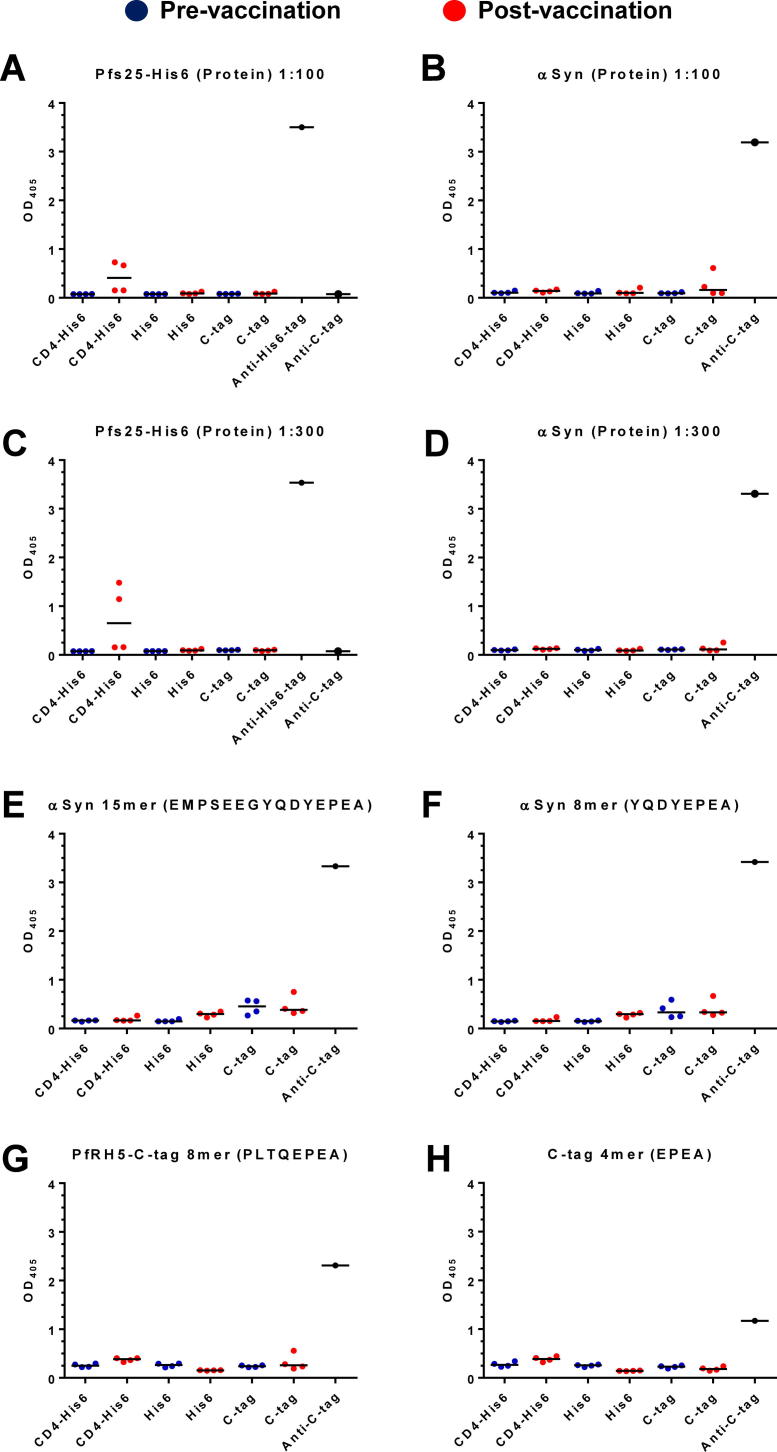
Assessment of ELISA responses against C-terminal tags. Rabbit sera were diluted 1:100 (and 1:300) and tested by ELISA against a panel of recombinant proteins and peptides for responses against the C-terminal purification tags. Sera were tested pre- and post-immunisation with *Plasmodium falciparum* reticulocyte-binding protein homolog 5 C-terminal hexa-histidine tag, C-terminal EPEA-tag (PfRH5-C-tag) and C-terminus of *P. falciparum* reticulocyte-binding protein homolog 5 fused to rat CD4 domains 3 and 4 followed by hexa-histidine tag (PfRH5-CD4d3 + 4-His6) formulated in Freund’s adjuvant. Control reagents against the hexa-histidine sequence (anti-hexa-histidine) and the EPEA C-tag (anti-C-tag) were included. Raw O.D. at 405 nm (OD_405_) data are shown plus medians for each group. (A, C) *P. falciparum* sexual-stage malaria antigen Pfs25 with a hexa-histidine tag (Pfs25-His6 protein); (B, D) α-synuclein protein; (E) 15 mer peptide and (F) 8 mer peptide corresponding to the C-terminal sequence of α-synuclein; (G) 8 mer peptide corresponding to the C-terminal sequence of PfRH5-C-tag; and (H) 4 mer EPEA (C-tag) peptide.

Finally, we assessed these rabbit sera for antibody responses against the C-terminal tags. Initially these were screened against an irrelevant protein with a His6 terminal tag (the sexual-stage malaria antigen Pfs25) (Li et al., 2016) and recombinant α-synuclein. Sera from rabbits immunised in our previously reported study were also included, where the C-terminus of PfRH5 protein was fused to rat CD4 domains 3 and 4 (CD4d3 + 4) followed by the His6 tag (Hjerrild et al., 2016). All rabbits were immunised three times with protein formulated in Freund’s adjuvant, and sera from pre- and post- vaccination were screened at 1:100 dilution. Modest anti-His6 responses were detected only in the rabbits immunised with PfRH5 protein fused to CD4d3 + 4-His6, but not when the His6 tag was used alone (Fig. 5A). All rabbits were negative against recombinant α-synuclein except one rabbit in the PfRH5-C-tag group where a low level response was observed (Fig. 5B). A repeat of this assay using sera diluted at 1:300 showed no detectable response (Fig. 5C, D). To investigate this further, the same sera were screened at 1:100 dilution using 15 mer and 8 mer peptides from the C-terminus of α-synuclein (Fig. 5E, F); an 8 mer peptide corresponding to the last four amino acids of PfRH5 followed by C-tag (Fig. 5G); as well as a 4 mer peptide for EPEA (Fig. 5H). All sera were negative against these peptides, whilst presence of the EPEA sequence was confirmed by use of the anti-C-tag single chain antibody reagent.

## 4 Discussion

In recent years, the PfRH5 antigen has emerged as a leading candidate for inclusion in next-generation vaccines aiming to prevent RBC invasion by blood-stage *P. falciparum* merozoites. However, our recent efficacy study in *Aotus* monkeys demonstrated that very high anti-PfRH5 IgG concentrations are still necessary to achieve significant control of blood-stage parasitemia (Douglas et al., 2015), although these and other data (Miura et al., 2009, Williams et al., 2012) suggest that IgG antibodies against full-length PfRH5 are more effective at inhibiting parasite growth on a per μg basis than those against other historically tested antigens. The need to achieve such high antibody responses by vaccination necessitates the production of a recombinant protein immunogen that can be formulated with a strong human-compatible adjuvant and progressed towards proof-of-concept clinical testing.

Production of full-length PfRH5 protein antigen initially proved very challenging, however substantial progress has been made in recent years using a variety of heterologous expression platforms including mammalian HEK293 cells (Crosnier et al., 2013), *E. coli* (Chiu et al., 2014, Reddy et al., 2014), baculovirus-infected insect cells (Patel et al., 2013, Chen et al., 2014) and a wheatgerm cell-free expression platform (Ord et al., 2012). Nevertheless, each of these platforms faces individual challenges for the cGMP production of a clinical vaccine batch – such as low yield, the necessary inclusion of C-terminal solubility tags that preclude use in humans (for example CD4d3 + 4), poor solubility, or lack of a scalable or cGMP-compliant process. Our recent identification of the ExpreS^2^ *Drosophila* S2 stable cell line system largely addressed these shortcomings, identifying a cGMP-compliant heterologous expression platform that could produce soluble full-length protein immunogen (Wright et al., 2014, Hjerrild et al., 2016). However, a very low overall process yield (typically <5% recovery of His6-tagged PfRH5 protein) meant the initial purification strategy was not suitable for scale-up and clinical biomanufacture of such a vaccine. Notably, the heaviest losses were observed during the initial capture step.

Here we investigated the use of a newly available CaptureSelect™ affinity purification method via C-terminal fusion of a four amino acid ‘C-tag’ to the PfRH5 antigen. Similar to our previous study (Hjerrild et al., 2016), a stable *Drosophila* S2 cell line was generated that secreted PfRH5-C-tag into the culture supernatant. We subsequently compared immunoaffinity purification using the C-tag versus an alternative IMAC step (using a cobalt-based TALON column) to capture His6-tagged PfRH5. Although both approaches showed a substantial improvement over our previously reported strategy (Hjerrild et al., 2016), the C-tag process consistently achieved >85% recovery and >70% purity in a single step purification directly from clarified, concentrated S2 cell supernatant under mild conditions. This purification process led to an overall process yield of 40–45%, very high product purity (>99%) and an overall yield of 1.8 mg/L from this polyclonal cell line, in contrast to an overall process yield of 25%, 85–90% purity and overall yield of 1.2 mg/L using the His6-tagged protein. Subsequent development of this process towards cGMP, including production of a high-expressing monoclonal S2 cell line, led to a further >10-fold improvement in overall process yield (Jin et al., unpublished data).

Biochemical and immunological characterization confirmed that the two proteins were equivalent – they both bound to the basigin receptor with the expected affinity and were recognised by a panel of eight mAbs, many of which bind to conformational epitopes (Douglas et al., 2014, Wright et al., 2014). Although purification tags may impact vaccine immunogenicity (Khan et al., 2012), the two versions of PfRH5 tested here performed similarly following immunisation of rabbits in terms of both quantitative and qualitative vaccine-induced IgG antibody responses. The PfRH5-specific IgG EC_50_s in the assay of functional GIA were also consistent with those we reported previously (Hjerrild et al., 2016). We also assessed immune responses against the C-terminal tags in immunised rabbits. In the case of the His6 tag, we could only detect responses when this was fused to PfRH5 in the context of a CD4d3 + 4-His6 construct (previously reported (Douglas et al., 2015, Hjerrild et al., 2016)), and not the direct fusion. Such anti-His6 responses have not been reported in humans, although this tag has been included in multiple candidate vaccines against malaria, some of which have proceeded as far as Phase IIb clinical trials in African children with no apparent safety concerns. In some cases, further aas have been present – totalling up to 29 non-pathogen aas fused to the desired antigen (Dutta et al., 2002, Angov et al., 2003, Otsyula et al., 2013).

In the case of the C-tag, we detected a very low level response in one rabbit against recombinant α-synuclein using a 1:100 serum dilution. This response titered out by 1:300 and was not also detectable using serum at 1:100 dilution against peptides corresponding to the C-terminus of α-synuclein or the PfRH5-EPEA fusion region. No responses were detected in any of the other rabbits. This marginal response in one animal suggests this four aa tag is very unlikely to induce antibodies that recognise human α-synuclein. Indeed, the sequence ‘EPEA’ is itself present in a number of pathogen proteins, and similar or longer regions of identity with human proteins are present by chance in licenced vaccine antigens. Less than 2% of linear epitopes recognised by mammalian antibodies are comprised of only four aas (Buus et al., 2012), whilst meta-analyses suggest that epitopes of antibodies for intact protein antigens comprise a minimum of 6–9 aa, with a mean of 15–20 (Kringelum et al., 2013, Stave and Lindpaintner, 2013), and with substantial dependence of binding affinity upon the conformation of the epitope.

Alpha-synuclein is predominantly intracellular and its precise function remains unknown, although in neural tissue it is believed to be involved in regulation of neurotransmitter release (Lashuel et al., 2013). Intracellular aggregates of α-synuclein, known as Lewy bodies, are implicated in Parkinson’s disease, however the binding of NbSyn2 has no apparent impact on the aggregation behaviour of α-synuclein (De Genst et al., 2010). It has also been suggested that antibodies against α-synuclein may play a protective role by promoting clearance of extracellular aggregates of the protein and lessening intracellular aggregation (Nasstrom et al., 2011, Yanamandra et al., 2011), with immunisation strategies being explored (Masliah et al., 2005). In contrast to these four aas, the 32 aa human tissue plasminogen activator (tPA) secretory peptide has also been used in numerous candidate vaccines produced at the University of Oxford, UK and subsequently tested in Phase I and II clinical trials in >3000 healthy adults and children: it has an excellent safety track record, with no detectable T cell response to the tPA sequence (Sheehy et al., 2011, Sheehy et al., 2012, Tameris et al., 2013, de Barra et al., 2014). Overall, the biology of α-synuclein makes it unlikely that antibodies to the EPEA tag, even if they should arise, would either reach the protein or be pathogenic. However, similar to any new vaccine technology, careful safety monitoring throughout Phase I studies and beyond would be paramount. In this regard, the CaptureSelect™ C-tag affinity resin, originally developed by BAC B.V. in the Netherlands (now Thermo Fisher Scientific), can be produced to be suitable for use in clinical biomanufacture. A VLP vaccine purified using this technology has recently entered Phase I/IIa clinical testing at the University of Oxford (Clinicaltrials.gov NCT02572388 and NCT02600975), and a PfRH5 protein vaccine (called RH5.1), also purified using the C-tag, has completed cGMP manufacture (Jin et al., unpublished data) and has entered early-phase clinical testing at the University of Oxford (Clinicaltrials.gov NCT02927145). These trials will provide the initial safety database in humans for the use of this small purification tag.

The challenges faced at the point of clinical translation of new concepts are numerous (Bregu et al., 2011). In many cases, a critical bottleneck is the substantial time and cost associated with the development of bespoke cGMP-compliant biomanufacturing processes, suitable for production of modest quantities of a novel product destined for proof-of-concept Phase I/II clinical trials. The C-tag technology has the potential to form the basis of a cGMP-compliant downstream purification process that is suitable as a pan-vaccine biomanufacturing platform, greatly improving the speed and ease with which novel protein-based products can progress to clinical testing.
